# Advanced and Invasive Cardiopulmonary Resuscitation (CPR) Techniques as an Adjunct to Advanced Cardiac Life Support

**DOI:** 10.3390/jcm11247315

**Published:** 2022-12-09

**Authors:** Manuel Obermaier, Stephan Katzenschlager, Othmar Kofler, Frank Weilbacher, Erik Popp

**Affiliations:** Department of Anaesthesiology, Heidelberg University Hospital, 69120 Heidelberg, Germany

**Keywords:** cardiac arrest, sudden cardiac death, emergency treatment, invasive procedures, heart massage, circulation, clamshell thoracotomy, extra-corporeal membrane oxygenation, echocardiography, resuscitative endovascular balloon occlusion of the aorta

## Abstract

Background: Despite numerous promising innovations, the chance of survival from sudden cardiac arrest has remained virtually unchanged for decades. Recently, technological advances have been made, user-friendly portable devices have been developed, and advanced invasive procedures have been described that could improve this unsatisfactory situation. Methods: A selective literature search in the core databases with a focus on randomized controlled trials and guidelines. Results: Technical aids, such as feedback systems or automated mechanical cardiopulmonary resuscitation (CPR) devices, can improve chest compression quality. The latter, as well as extracorporeal CPR, might serve as a bridge to treatment (with extracorporeal CPR even as a bridge to recovery). Sonography may be used to improve thoracic compressions on the one hand and to rule out potentially reversible causes of cardiac arrest on the other. Resuscitative endovascular balloon occlusion of the aorta might enhance myocardial and cerebral perfusion. Minithoracostomy, pericardiocentesis, or clamshell thoracotomy might resolve reversible causes of cardiac arrest. Conclusions: It is crucial to identify those patients who may benefit from an advanced or invasive procedure and make the decision to implement the intervention in a timely manner. As with all infrequently performed procedures, sound education and regular training are paramount.

## 1. Introduction

The cornerstones of cardiopulmonary resuscitation (CPR) as we know it today have been laid in 1960 through the revolutionary principle of closed-chest cardiac massage introduced by Kouwenhoven, Jude, and Knickerbocker [1], and the pioneering works on artificial respiration by Safar [2]. Six decades later, and despite major efforts in research and all advancements in technology, little has changed in the poor prognosis of patients suffering sudden cardiac arrest [3].

In order to remedy this highly unsatisfactory situation, clinician scientists worldwide strive to enhance treatment and markedly improve survival. Some of these supposedly revolutionary treatment approaches had to be abandoned again due to lack of efficacy [4], while others are now an integral part of the international guidelines concerning CPR [5]. In addition to various educational concepts and abundant awareness campaigns which address the broad public [6], some most recent developments concerning advanced therapeutic options focus on hyperinvasive strategies and approaches [7]. It is recognized that maintaining blood pressure targets during cardiac arrest and, after the return of spontaneous circulation (ROSC), affects survival rates [8,9]. As Brede concisely determines in a current editorial, each “long term survival” is preceded by a ROSC and, therefore, “all potential adjunct treatments to increase the rate of ROSC should be assessed” [10].

In cases where ROSC cannot be achieved immediately, chest compression continuity and its quality are key determinants for survival in cardiac arrest [11,12]. Conventional manual chest compressions are demanding, leading to increased interruptions [13], and are insufficiently performed [14] due to rescuers’ exhaustion. Subsequently, no- and low-flow times are increasing which affects the hemodynamic situation [15]. This is considered a main determinant that prevents ROSC and, subsequently, survival due to impaired myocardial and brain perfusion [16,17].

There are several advanced and invasive techniques readily available in hospital emergency departments, which seem to stay unused in the out-of-hospital emergency setting. Consequently, in a new approach, the attempt is made to bring these techniques, devices, and qualified personnel who routinely apply these techniques in an in-hospital cardiac arrest (IHCA) setting to patients suffering an out-of-hospital cardiac arrest (OHCA) [18,19].

We aim to provide a narrative overview of currently discussed advanced principles and invasive techniques as adjuncts to advanced life support (ALS).

## 2. Methods

We conduct a selective literature search in established scientific databases as well as preprint servers, clinical trials registry platforms (Tables S1 and S2), and internet search engines for publications with a focus on randomized controlled trials (RCT), recommendations from scientific societies and official guidelines concerning advanced and invasive technical therapeutic options in the treatment of cardiac arrest.

We define those resuscitation techniques as “advanced and invasive” that go beyond routine application under the “standard” ALS algorithm.

## 3. Results and Discussion of Advanced and Invasive Techniques

As with any complex and seldomly utilized technique, situation awareness, sound education, and continuous training in technical and non-technical skills are paramount. The basis for all advanced resuscitation measures is the uninterrupted and effective basic resuscitation that has been started as quickly as possible [6,11]. This is crucial for patient survival and often advanced and invasive techniques are not useful unless lay resuscitation has taken place [5,20]. Consequently, every person working in the medical field should regularly participate in CPR training courses according to his or her qualifications.

There is a broad array of courses offered for learning and training in advanced invasive procedures that go beyond the “standard” ALS, as well as sonography and non-technical skills, such as crisis resource management [21,22,23]. In addition, cognitive aids can help to work through complex situations in a structured way [24]. It must be emphasized that all trials discussed in this work refer to well-trained and experienced experts in specialized centers. This level cannot usually be achieved by attending courses alone, but also requires regular work in a center with a correspondingly high number of cases.

### 3.1. Real-Time Audio or Audiovisual CPR Feedback Devices

Considerations that poor CPR quality with frequent interruptions may entail low survival rates [25,26], as previously stated, led to the development of feedback devices that are designed to measure important CPR quality parameters (i.e., compression depth, relief, frequency, and hands-off time) and give real-time audio (clickers for tactile feedback) or audiovisual feedback. This enables life support providers to continuously monitor and, when appropriate, to self-adjust their external chest compressions in real-time. Furthermore, most systems allow the users to retrospectively analyze the performance for educational purposes. Therefore, real-time feedback devices are expected to improve CPR quality and possibly patient survival [27].

This review focuses on audio or audiovisual feedback devices but does neither address functions such as metronome sound, which occasionally are referred to as feedback devices [11], nor software applications for mobile communication devices.

Several trials report partially contradictory or ambivalent results concerning the effect of real-time feedback systems on CPR quality and patient outcome (Table 1) [27,28]. In addition to several observational studies, mainly from simulated training scenarios, only two RCTs could be identified. An individualized RCT and its secondary analysis evaluating an audio-only feedback device in a hospital setting found better adherence to resuscitation guidelines, as well as significantly higher rates of ROSC and survival until intensive care unit (ICU) and hospital discharge [29,30]. A large cluster RCT evaluating an audiovisual device in an out-of-hospital setting confirms the findings regarding improved CPR quality, but this was not associated with improved survival rates [31]. This result calls into question the previously assumed clinical relevance of improved chest compressions.

There seems to be sufficient objective evidence that the application of real-time audio or audiovisual feedback devices contributes to an improvement of chest compression quality and continuity, as laid down in resuscitation guidelines. In order to either confirm or refute the hypothesis that improved chest compressions actually lead to higher survival rates with favorable neurological outcomes, further well-designed, sufficiently powered, and properly conducted studies are necessary.

Nevertheless, currently, valid guidelines recommend the use of real-time audiovisual feedback devices in order to improve CPR quality as a part of a comprehensive quality improvement program [11,32].

### 3.2. Automated Mechanical CPR (mCPR)

Whilst feedback devices might contribute to improving chest compression quality, they cannot resolve the problem of increasing exhaustion of staff members during prolonged CPR attempts or the impaired quality of chest compression in certain situations (i.e., on transport). Automated mCPR devices have been developed for this purpose and perform consistent chest compressions, either by a semi-circumferential load-distributing band or a vertical piston. It is a matter of in-depth discussion on whether their application might improve patient survival rates (Table 2).

#### 3.2.1. Rationale

A large RCT, the ASPIRE trial was terminated prematurely by the data and safety monitoring board, after there was—despite similar survival rates at 4 h after the incident—a trend to impaired survival until hospital discharge and neurologic state [33]. Despite slightly distinctive definitions of ROSC, none of the other studies revealed any statistically significant different rates of ROSC at any time or sustaining ROSC until hospital admission, respectively. The CIRC trial, an RCT that compares an automated mCPR device utilizing a load-distributing band to conventional manual CPR, found a slightly diminished rate of sustaining ROSC (odds ratio (OR) 0.84, 95% confidence interval (CI) 0.73–0.96) and survival after 24 h (OR 0.86, 95% CI 0.74–0.998) in the mCPR group. This observation is recouped during the stay in the hospital, as the rate of survival until hospital discharge (OR 0.89, 95% CI 0.72–1.10) and neurologic state at discharge according to the modified Rankin Scale (mRS) (OR 0.80, 95% CI 0.47–1.37) do not show any statistically significant differences [34]. Two other well-conducted multi-center RCTs, the LINC trial [35] and the PARAMEDIC trial [36], found equal survival rates until hospital discharge. In the latter, this was associated with a slightly but significantly worse neurologic outcome in the intervention group receiving automated mechanical chest compressions through a vertical piston device. Adjusted OR for the risk of favorable neurologic outcome (defined as CPC 1–2) was 0.72 (95% CI 0.52–0.99) [36]. Apart from these exceptions, no statistically significant differences in survival rates and neurological outcomes were observed in any of the large RCTs [34,35,36,37]. In the as-treated analysis, the MECCA investigators found improved survival rates compared to conventional manual CPR, if the automated mCPR device utilizing a vertical piston was attached “early” during the course, viz., before moving the patient to the ambulance [37]. In all these studies, it is of key importance to consider that the results and, thereby, the conclusions of superiority or inferiority of any technique mainly depend on the quality of conventional manual CPR delivered in the respective control groups [38,39].

#### 3.2.2. Significance of the Time Aspect

The observations regarding the importance of time correspond with observations from a retrospective registry analysis, where a higher rate of ROSC was found in the mechanical CPR groups, which is mainly attributable to lower risk factors among the population within this group. Compared with the predicted rate of ROSC (the ROSC after cardiac arrest (RACA) score), patients in the load-distributing band group achieved ROSC significantly more frequently (predicted 46.6%; achieved 57.1%, 95% CI 49.5–64.5%), while patients in the mechanical piston group (predicted 43.4%; achieved 46.5%, 95% CI 38.4–54.7%) and manual CPR group (predicted 39.9%; achieved 40.1%; 95% CI 39.2–41.1) only fulfilled the predicted outcome. Adjusted for epidemiologic and therapeutic factors, OR for ROSC was 1.70 (95% CI 1.12–2.57) for the load-distributing band system and 1.66 (95% CI 1.09–2.51) for the mechanical piston system [40]. On the contrary, in a pilot study, which neither excluded resuscitations shorter than 5 min nor considered CPR duration in its regression analysis, we found an OR for ROSC of 0.82 (95% CI 0.64–1.07) for the load-distributing band system and 0.48 (95% CI 0.36–0.64) for the mechanical piston system [41]. This is consistent with a secondary analysis of the CIRC trial utilizing a logistic regression analysis that identified an increased probability for survival until hospital discharge after automated mCPR (OR 1.46; 95% CI 1.03–2.07) if adjusted for emergency medical service (EMS) response time [42]. In the original publication of the CIRC trial, response times > 16 min have been excluded [34]. A longitudinal, phased cohort study from Singapore shows a higher rate of survival until hospital discharge in the load-distributing band mCPR group (8.1% vs. 1.9% in the manual CPR group) if the EMS response time was < 8 min. If response times were > 8 min, only a few patients survived in both groups [43].

**Table 2:** Overview of selected studies on automated mechanical vs. conventional manual chest compression.

Trial Abbreviation and Quotation	Design and Inclusion Criteria	Main Findings and Limitations
(a) RCTs on automated mCPR vs. conventional manual chest compression		
ASPIRE [33]	mCPR (AutoPulse^®^) vs. conventional manual CPR; prospective cluster RCT, multi-center (Canada, USA); out-of-hospital; 2004–2005; 394 vs. 373 cases out of 1377 Inclusion criteria: age ≥ 18 and non-TCA	Stopped by DSMB, ±survival (after 4 h), ↓ discharge, ↓ CPC Limitations: design (allocation concealment and not powered for secondary analyses), selection bias (trial vehicles), performance bias (device implementation, noncompliance, quality monitoring), training effect, and COI (funding by manufacturer)
CIRC [34]	mCPR (AutoPulse^®^) vs. conventional manual CPR; prospective individually RCT, multi-center (USA, Austria, Netherlands); out-of-hospital; 2009–2011; 2099 vs. 2132 cases out of 4753 Inclusion criteria: age ≥ 18, presumed cardiac origin (non-TCA), and response time ≤ 16 min	↓ “sustained” ROSC, ↓ survival (after 24 h), ±discharge, ±CPC Limitations: selection bias (response time), performance bias (guideline revision, quality monitoring), reporting bias (post-randomization exclusions), training effect, and COI (co-author is an employee of the manufacturer)
LINC [35]	mCPR (LUCAS^®^) with simultaneous defibrillation vs. conventional manual CPR with sequential defibrillation; prospective individually RCT, multi-center (Sweden, UK, Netherlands); out-of-hospital; 2008–2013; 1300 vs. 1289 cases out of 4998 Inclusion criteria: age ≥ 18, non-TCA, and no defibrillation before the device arrived on scene	±ROSC, ±admission, ± survival (after 4 h and 1 and 6 months), ±discharge, ±CPC Limitations: performance bias (defibrillation simultaneous vs. sequential, guideline revision, quality monitoring, noncompliance), training effect, and COI (device developed by investigating university)
PARAMEDIC [36]	mCPR (LUCAS^®^) vs. conventional manual CPR; prospective cluster RCT, multi-center (UK); out-of-hospital; 2010–2013; 1652 vs. 2818 cases out of 4689 Inclusion criteria: trial vehicle first on the scene, age ≥ 18, and non-TCA	±ROSC, ±admission, ±survival (after 1, 3 & 12 months), ↓ CPC Limitations: design (allocation concealment and the sample size was increased), selection bias (trial vehicles), performance bias (guideline revision, quality monitoring), and training effect
MECCA [37]	mCPR (LUCAS^®^) vs. conventional manual CPR; prospective cluster RCT, multi-center (Singapore); out-of-hospital; 2011–2012; 302 vs. 889 cases out of 1274 Inclusion criteria: age ≥ 21, presumed cardiac entity (non-TCA), and attended to by ambulance crew	±ROSC, ±survival (after 24 h and 30 days), ±discharge; as-treated analysis: any outcome ↑ if mCPR device attached early Limitations: design (not powered for secondary analyses and allocation concealment), performance bias (quality monitoring and noncompliance), training effect, and COI not reported
(b) Non-RCTs on automated mCPR vs. conventional manual chest compression		
German Resuscitation Registry [38,40]	mCPR (LUCAS^®^, AutoPulse^®^) vs. conventional manual CPR; retrospective registry analysis, multi-center (Germany); out-of-hospital; 2007–2014; 912 vs. 18,697 cases out of 35,593 Inclusion criteria: cases documented in the registry, age ≥ 18, and non-TCA	↑ (AutoPulse^®^) or ± (LUCAS^®^) ROSC (if CPR duration considered, ±(AutoPulse^®^) or ↓ (LUCAS^®^) with general application) Limitations: design (retrospective), selection bias (voluntary participation in the registry), performance bias (registry data, quality of documentation, and voluntary), and loss of follow-up
LDB device for OHCA resuscitation [43]	mCPR (AutoPulse^®^) vs. conventional manual CPR; prospective phased longitudinal observational cohort study, single-center (USA); out-of-hospital; 2001–2005; 284 vs. 499 cases out of 2294 Inclusion criteria: age ≥ 18 and non-TCA	↑ ROSC, ↑ admission, ↑ discharge, ±CPC, few survivors if response time > 8 min Limitations: design (observational), performance bias (hypothermia and device implementation), training effect, and COI (funding by manufacturer, co-author is an advisor for the manufacturer)

Legend: ± = equal, comparable, and no statistically significant difference; ↑ = more, higher, better, and superior; ↓ = less, lower, worse, and inferior. Abbreviations: RCT = randomized controlled trial, CPR = cardiopulmonary resuscitation, mCPR = (automated) mechanical CPR, DSMB = data and safety monitoring board, CPC = Glasgow–Pittsburgh cerebral performance category, ROSC = return of spontaneous circulation, COI = conflict of interest, TCA = traumatic cardiac arrest, and OHCA = out-of-hospital cardiac arrest.

The installation of automated mCPR devices may delay the time until the first defibrillation, depending on which source is consulted, for a period of up to 2.1 min [33,34,35,38]. A Cochrane review suggests that these negative impacts on CPR quality, namely lag-times until the application of the device with increased hands-off time and delay of the first defibrillation in shockable rhythms, may negate “any physiologic benefit observed in preclinical studies” [39]. Nevertheless, defibrillation is feasible and safe during ongoing automated mechanical chest compressions and, thereby, reduces the hands-off time during the later course [5,35].

#### 3.2.3. Decision Criteria

Further data regarding secondary outcomes, such as long-term survival (e.g., after 1, 3, 6, or 12 months) or CPR quality surrogates (e.g., compression depth and frequency, hands-off time, injuries, blood pressure, coronary or cerebral perfusion pressures, respiratory, metabolic, and other laboratory parameters) show heterogeneous and, in parts, contradictory results. The later the endpoints, the slighter the difference between mechanical and manual CPR outcomes—a frequent observation within longitudinal resuscitation studies [38,39,44,45]. Trauma due to chest compression may occur both after conventional manual and automated mCPR in the same frequency, but the injury patterns seem to differ. Patients who received conventional manual chest compressions typically show anterior rib fractures, sternum fractures, unshaped midline chest abrasions along the sternum, visceral bleeding, and retrosternal hematoma. In contrast, patients who received automated mechanical chest compressions show posterior rib fractures, vertebral fractures, shaped skin abrasions along the anterolateral chest and shoulder, visceral bleeding including liver and splenic lacerations, and retroperitoneal hematoma [46,47,48,49].

Automated mCPR cannot reverse any cause of cardiac arrest, nor does it lead to higher survival rates compared to manual CPR. Hence, manual chest compression should be the standard technique, but under special circumstances and in particular fields of application, the devices may provide effective and safe chest compressions until an adequate health care facility is reached, where the definitive treatment can be performed. Thus, these systems may be beneficial as a bridge to treatment tool or as a bridge to decision on further treatment, and may be considered especially during prolonged resuscitation attempts. This might arise under specific conditions, such as, for example, sustaining ventricular fibrillation (VF) [50] or hypothermic cardiac arrest [50,51,52,53,54], patient transportation [5,55,56,57], diagnostics, such as computed tomography (CT) [5,58,59,60] (not chest X-rays [61]), and interventions, such as percutaneous coronary intervention (PCI) [5,58,62], fibrinolysis [60,63], dialysis [54], extra-corporeal membrane oxygenation (ECMO) [5], transcatheter aortic valve implantation (TAVI) [64], surgery [65,66], or organ preservation until retrieval [5,67]. On the other hand, after the implementation of mechanical CPR devices in a German EMS system, a dramatic increase in transportation with ongoing CPR, even for patients with unfavorable prognoses, has been observed [68].

#### 3.2.4. Therapeutic Strategy

Thus, appropriate patients with potentially reversible and, therefore, treatable causes of cardiac arrest should be carefully identified and selected, as they might profit from transportation to a suitable hospital under ongoing automated mCPR with clear therapeutic approaches [54]. This interpretation is consistent with the manufacturers’ original intention, as laid down in the 1979 US Food and Drug Administration (FDA) classification report: “(…) the device is not designed to replace manual CPR. The literature seems to recommend it for certain situations (…)” [69]. Currently, valid guidelines recommend trained teams that are familiar with the device to consider automated mCPR “only if high-quality manual chest compression is not practical or compromises provider safety” [5], and under special circumstances, such as hypothermia, metabolic disorders, low cardiac output state, obesity, the need of prolonged transportation, difficult terrain, and restricted space conditions, for example, helicopter flights due to a limited cabin size [54].

### 3.3. Extra-Corporeal Cardiopulmonary Resuscitation (eCPR)

As previously stated, it is necessary to keep the low-flow time during CPR as short as possible. In the setting of an OHCA, it is essential to identify patients early who could benefit from an advanced or invasive procedure, such as extra-corporeal life support (ECLS), often synonymously referred to as extra-corporeal CPR (eCPR). As this approach involves a specialized multidisciplinary team, the decision should be made involving all parties.

#### 3.3.1. Rationale

eCPR has proven to increase the chances of survival with good neurological outcomes in patients with refractory cardiac arrest treated in experienced centers by expert teams. Two randomized controlled trials demonstrated the effect of eCPR and a subsequent invasive diagnostic and treatment strategy, such as PCI. Early invasive treatment is the cornerstone of increased survival in these patients (Table 3). Bělohlávek et al. demonstrated in a secondary analysis a favorable neurological outcome in 22% of patients who received eCPR, which is the same proportion as patients in the standard-of-care group. Compared with 31.5% in their intervention group, this demonstrates the effect of an early invasive treatment strategy even without eCPR [7]. A smaller phase 2 RCT, the ARREST trial, was stopped early by the DSMB, as superiority exceeded the prespecified monitoring boundary in a planned interim analysis. The survival rate in the eCPR group was significantly higher than in the control group, but the case number was too small to demonstrate benefits in neurological outcomes [70]. In a secondary analysis from the original Prague OHCA trial, Rob et al. found a significantly higher rate of survivors in patients without prehospital ROSC and eCPR compared to standard ACLS. Furthermore, a good neurological outcome (CPC 1 or 2) was only achieved by 1.2% of patients treated with standard ACLS without prehospital ROSC. This compares to 21.7% in the eCPR group and 56.6% when prehospital ROSC was achieved [71]. All studies that are assessing the effect of eCPR in patients with OHCA have a high rate of bystander CPR. Although it should not be limited to this factor, bystander CPR has been shown to be crucial in all OHCA cases [72].

Currently, there is no consensus on when eCPR should be performed. Some studies suggest that the latest decision point should be around 30 min after cardiac arrest [73]. Recent studies in Prague [7] and Denmark [74] showed a beneficial effect in patients with > 30 min of low-flow time. Furthermore, the study by Mørk et al. showed a neurologic intact survival in 20% of the patients receiving eCPR with a low-flow time of >75 min [74]. In contrast, earlier studies have shown no benefit when eCPR was initiated after 60 min of cardiac arrest [73].

**Table 3:** Overview of selected studies on eCPR alone or as a part of a combined hyperinvasive approach vs. conventional manual chest compression.

Trial Abbreviation and Quotation	Design and Inclusion Criteria	Main Findings and Limitations
(a) RCTs on eCPR vs. conventional manual chest compression or automated mCPR		
ARREST [70]	eCPR vs. conventional manual CPR or automated mCPR in patients with VF; prospective individualized RCT, single-center (USA); emergency department; 2019–2020; 15 vs. 15 patients out of 36 inclusion criteria: age 18–75, initially documented OHCA rhythm VF or pulseless ventricular tachycardia, no ROSC following three defibrillations, body morphology allows mCPR, and estimated transfer time < 30 min	Stopped by DSMB, superiority exceeded prespecified monitoring boundary; ↑ discharge, ↑ survival (after 3 and 6 months), ±CPC Limitations: design (single-center), performance bias (high eCPR expertise), low number of participants since stopped early, and training effect
(b) RCTs on a combined hyperinvasive approach vs. conventional manual chest compression		
Prague OHCA study [7]	mCPR (LUCAS^®^), early intra-arrest transport, eCPR, invasive assessment and treatment vs. conventional manual CPR; prospective individually RCT, single-center (Czech Republic); out-of-hospital; 2013–2020; 124 vs. 132 cases out of 256 Inclusion criteria: age 18–65, witnessed collapse, presumed cardiac cause, ≥ 5 min ALS without sustained ROSC, unconsciousness (Glasgow Coma Score < 8), eCPR team, and ICU bed capacity available	Stopped by DSMB, possibly underpowered; ± “sustained” ROSC, ± “neurologic recovery” [survival with CPC 1–2] (↑ after 30 days, ± after 180 days), ± “cardiac recovery” Limitations: design (single-center, limited enrollment, power, and crossover), performance bias (high eCPR expertise and noncompliance), selection bias (high bystander CPR rates), and training effect
(c) retrospective cohort studies on mechanical circulatory support vs. mCPR		
Survival and neurological outcome after OHCA treated with and without mechanical circulatory support [74]	eCPR (with or without Impella) vs. mCPR; retrospective cohort study, single-center (Denmark); emergency department; 2015–2019; 101 vs. 216 cases out of 1015 Inclusion criteria: age ≥ 18 and transport to the hospital with refractory OHCA	↑ ICU admission, ↑ discharge, ↑ survival (after 30 days and 1 year), ↑ CPC Design (retrospective), selection bias (voluntary participation in the registry), performance bias (registry data, quality of documentation, and voluntarily), and loss of follow-up

Legend: ± = equal, comparable, and no statistically significant difference; ↑ = more, higher, better, and superior; ↓ = less, lower, worse, and inferior. Abbreviations: RCT = randomized controlled trial, CPR = cardiopulmonary resuscitation, eCPR = extracorporeal CPR, mCPR = (automated) mechanical CPR, VF = ventricular fibrillation, DSMB = data and safety monitoring board, CPC = Glasgow–Pittsburgh cerebral performance category, OHCA = out-of-hospital cardiac arrest, ALS = advanced life support, ROSC = return of spontaneous circulation, and ICU = intensive care unit.

#### 3.3.2. Decision Criteria

As important as the optimal timing of eCPR cannulation are the decision criteria for eCPR initiation. National [75] and international [5,20] guidelines are available to answer this question. Overlapping inclusion criteria are younger age, witnessed arrest, duration of no-flow time <5 min, and time until eCPR initiation <60 min. Further criteria to consider are signs of life under CPR [76], intermittent ROSC or recurrent VF, neuroprotective circumstances, availability of cardiac arrest center with PCI capability, blood gas analysis, known diseases and, if known, the patient’s request. Signs of life under CPR were independently associated with a favorable neurological outcome. In a study analyzing 434 individuals undergoing eCPR, any sign of life before or throughout CPR was associated with an OR for a favorable neurological outcome of 7.35 (95% CI 2.71–19.97). This was further assessed for different types of signs of life, such as gasping, pupillary light reaction, and increased level of consciousness. Those signs had an OR of 1.75 (95% CI 0.95–3.21), 5.86 (95% CI 2.28–15.06), and 4.79 (95% CI 2.16–10.63), respectively. Patients with pulseless electrical activity (PEA) or asystole had a 12% (95% CI 5–25) chance of 30-day survival with CPC 1–2 when any sign of life was present. In contrast, patients without signs of life in PEA or asystole had a 0% (95% CI 0–7%) 30-day survival with CPC 1–2. This effect is also seen in patients with a shockable rhythm, where 23% (95% CI 17–30%) with any sign of life and only 4% (95% CI 1–11%) without any sign of life had a good neurological outcome, respectively [76].

#### 3.3.3. Cannulation

It remains unclear which cannulation strategy is the best for eCPR in OHCA patients. Kashiura et al. demonstrated that patients who underwent eCPR cannulation with both ultrasound guidance and fluoroscopy had an independently associated lower complication rate compared to ultrasound guidance alone (adjusted OR 0.14, *p* = 0.024). Furthermore, the time until eCPR started was the same in both groups, with a median of 17 min [77].

This compares to a mean cannula insertion time of 22.5 ± 9.9 min when a hybrid cutdown technique is performed by non-surgeons [78]. For all three approaches, failure rates were low with a change to surgical approach in four cases (8%) if ultrasound was the only method used. The overall failure rate for the hybrid approach was as low as 7.4%.

Danial et al. assessed the impact of cannulation techniques in 814 patients. Until November 2016, all patients were cannulated using a surgical approach, while hospital policy changed in November 2016 and a percutaneous approach was used from there on. Using this retrospective data, a propensity score-matched cohort study for cannulation techniques in 532 veno-arterial ECMO patients was performed. Patients were divided into a surgical and percutaneous group, with 266 patients each. Compared to a surgical approach, percutaneous access, performed via doppler ultrasound, had a higher 30-day overall survival rate (63.8% vs. 56.3%, *p* = 0.034) and more vascular complications after cannula removal (14.7% vs. 3.4%, *p* < 0.001), which mainly resulted in surgical revision for persistent bleeding. Furthermore, a lower infection rate at the cannulation site was recorded (16.5% vs. 27.8%, *p* = 0.001) [79].

Especially if a contralateral approach is used, the risk of arterio-arterial or veno-venous cannulation is given; this can be limited with ultrasound guidance.

#### 3.3.4. Therapeutic Strategy

Similar to automated mCPR, eCPR should not be considered a definitive treatment in cardiac arrest, but as a bridge to decision or bridge to treatment while organ perfusion (particularly cerebral and coronary) is maintained. Moreover, eCPR might have the potential to serve as a bridge to recovery. Again, it is crucial to identify patients with potentially reversible entities of cardiac arrest who might profit from this invasive treatment; for example, therapy-refractory ventricular fibrillation or severe hypothermia. In-depth expertise is essential for the implementation of this measure, which requires comprehensive technical and non-technical skills and correspondingly high case numbers at the institutions providing the training. In addition to human resources, the receiving hospital must also have the equipment and capacity for this highly critical patient population. Moreover, it requires a system with highly skilled care providers, as well as the equipment and capacity necessary to provide adequate continuing care for patients.

In addition, eCPR might also serve as a bridge to donation. Although this should never be the main purpose of initiation, it can be a secondary benefit to others when, despite the best effort, ECMO weaning is not possible or severe brain damage has already occurred.

### 3.4. Sonography

Sonography (point-of-care ultrasound, POCUS) is increasingly becoming a key skill for the evaluation of CPR quality, reversible causes of cardiac arrest, and hemodynamic situations after sustained ROSC. Despite all the versatility of its applications, to date, no RCT has demonstrated an improvement in patient outcomes due to the performance of sonography and the resulting therapeutic consequences.

There are an increasing number of courses to learn emergency ultrasonography, although its performance under emergency conditions and, especially, during ongoing CPR, requires appropriate experience to obtain reliable images and draw clinical conclusions from it.

The extended Focused Assessment with Sonography for Trauma (eFAST) protocol has been established for the rapid assessment of critically injured patients [80]. This protocol specifies defined ultrasound positions with a focus on the detection of pericardial effusion, pneumothorax, and free abdominal and thoracic fluid, which are considered potentially reversible causes of cardiac arrest [5]. In addition, the Focused Echocardiographic Evaluation in Life Support (FEEL) protocol has been established to address cardiac causes of hypotension and cardiac arrest by basic trans-thoracic echocardiography (TTE) [81]. Using these protocols has the potential to detect reversible causes of cardiac arrest and improve hemodynamic therapies in patients during shock. Although the importance of early detection of reversible causes of circulatory arrest seems obvious, evidence of improved survival rates based on ultrasonography and the resulting therapeutic consequences is still lacking.

Accordingly, current guidelines do not state at which time in a cardiac arrest case ultrasound should be used. Guidelines from the European resuscitation council explicitly say that only experienced providers should use ultrasound in emergency situations [32]. This increases image quality and the ability to draw therapeutic conclusions from it. In order to minimize hands-off time, the ultrasound probe should be placed at the subxiphoidal position during ongoing chest compressions. One team member, who is not responsible for the conduction of the ultrasound exam, should count from five backward and chest compressions should be resumed automatically. Image storage can be used if reassessment is necessary.

There are cases where TTE is not possible or visualization is severely limited. In such cases, trans-oesophageal echocardiography (TOE) can be used to identify reversible causes of cardiac arrest with ongoing chest compressions.

#### 3.4.1. Optimisation of Chest Compressions

**Figure 1:**
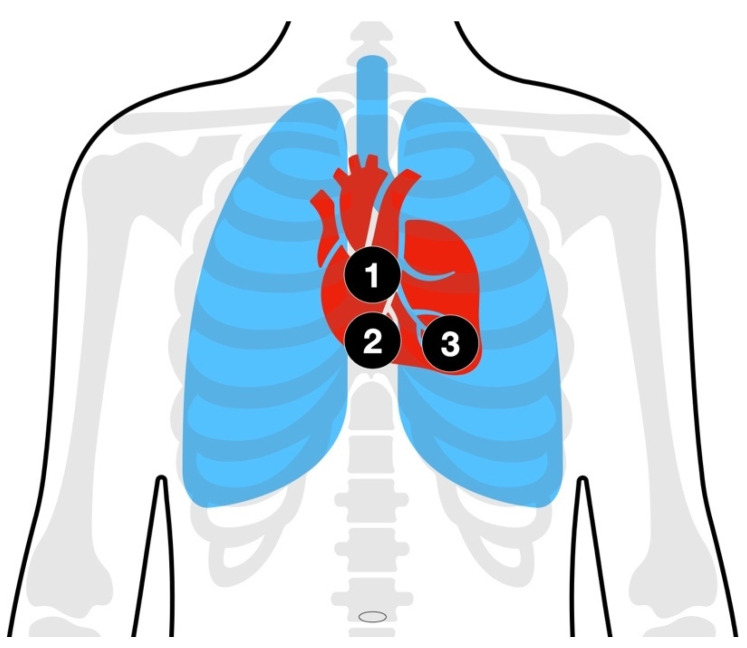
Schematic diagram of chest compression points. (**1**) “classic” hand position according to current guidelines [11,32]; (**2**) a more caudal approach with the hand still on the sternum; (**3**) a more caudal and left lateral approach in order to provide the maximum compression upon the left ventricle (LV).

High-quality chest compression is the cornerstone in the treatment of each cardiac arrest case. The “middle of the chest” has been proven to be an unreliable place when the left ventricle (LV) should be compressed [82,83,84]. One approach would be to continuously assess the capnography waveform and change hand positions based on three pre-defined positions (Figure 1).

As this “blind” approach can be time-consuming and waveform capnography can be impaired due to various circumstances, such as the underlying pathology, echocardiography during CPR is a powerful tool for the detection of the area of maximal compression (AMC), and following the optimization of chest compression.

Although availability can be limited, the TOE probe can be inserted during ongoing chest compressions. Assessment of the AMC is performed in the midoesophageal 4-chamber view (ME4CH) and the midoesophageal long axis (MELAX). As soon as the AMC is identified over the left ventricular outflow tract (LVOT) or the right ventricle (RV) (Video S1), hand positioning should be optimized. Continuous assessment of the AMC is now possible while maintaining high-quality chest compressions.

Blaivas described the performance of TOE under CPR in 2008 with a case series from an emergency department highlighting the benefits of continuous chest compressions while seeking reversible causes that led to cardiac arrest [85].

A 4-view approach was used in an emergency department in a prospective observational study. Teran et al. performed four standardized TOE views: the ME4CH, MELAX, midoesophageal bicaval (ME Bicaval), and transgastric short axis (TGSAX). Out of 33 total cases with OHCA, they were able to assess the AMC in 17 patients during ongoing CPR, while the other 16 patients achieved ROSC before resuscitative TOE was conducted. TOE was performed within 12 min (SD 8.16) after the patient’s arrival. This revealed nine cases (53%) with an AMC over the LVOT or aortic root. Changes in the compression position resulted in an observable improvement of the end-tidal carbon dioxide partial pressure (etCO_2_) and perfusion. Although TOE identified RV dilation in thirteen patients (39%), pulmonary embolism (PE) was only suspected in two cases. This is in line with the negative association of ROSC and intra-arrest RV dilation with an OR of 0.7 (95% CI 0.01–0.82). Although no benefit regarding favorable neurological survival was found when AMC was adjusted, TOE influenced clinical management with regard to diagnostic, therapeutic, and prognostic consequences in 97% (32/33) of the cases [86]. Further studies should focus on the change in etCO_2_ and hemodynamic parameters when AMC is adjusted.

#### 3.4.2. Detecting and Addressing Reversible Causes of Cardiac Arrest

Hypovolaemia: In traumatic cardiac arrest, hypovolaemia due to exsanguination is one of the most common reversible causes, and invasive therapeutic options are available. In order to enhance certainty before performing invasive interventions, the eFAST protocol can be applied to detect free abdominal or thoracic fluid. Venous congestion during CPR can limit the significance of inferior vena cava (IVC) assessment. Current guidelines state limited knowledge about the use of ultrasound for the detection of hypovolemia in cardiac arrest [5,32].

Thromboembolism: Thrombosis, either cardiac or pulmonary, is one of the most frequent causes of sudden cardiac death. As PE has non-specific clinical signs and symptoms that may lead to PEA, it is crucial to look for specific echocardiographic signs before the patient further deteriorates [87,88]. Therefore, visual assessment should be the main priority, as this can be performed in every setting with a handheld ultrasound device.

In patients with cardiac arrest, RV dilation is a common finding, especially if resuscitation has been ongoing for a prolonged period [89]. This weak association between RV dilation and PE brings a challenge to the table, leaving only reduced possibilities of identifying PE during CPR. Right heart mobile thrombus could be seen during CPR, suggesting the presence of PE [88]. A second finding, deep vein thrombosis (DVT), can be detected in about 30–50% of patients with PE by compression ultrasound [90].

While the need for emergent ultrasound diagnostics is not given in shockable rhythms, it may provide valuable information in peri-arrest situations or following ROSC, if a 12-lead electrocardiogram is inconclusive. Regional wall motion abnormalities can be seen even in patients without significant repolarisation abnormalities.

Tension pneumothorax: Ultrasound is not only faster than conventional chest X-rays or CT—it also offers better sensitivity and specificity than chest X-rays. In addition, it can be performed directly at the site of emergency [91] if the “classical” diagnostic means are uncertain.

Pericardial tamponade: A pericardial effusion was previously considered only a suspected diagnosis in the out-of-hospital setting, which could not be ruled out with certainty. With the increasing availability of portable ultrasound equipment, even users with little experience can answer the question of the presence of a pericardial effusion using the eFAST or FEEL protocol in the field [92].

### 3.5. Resuscitative Endovascular Balloon Occlusion of the Aorta (REBOA)

The principle of intra-aorta balloon catheter tamponade dates back to the 1950s and has first been described in soldiers with battle-related severe abdominal and pelvic bleeding during the Korean War [93].

**Figure 2:**
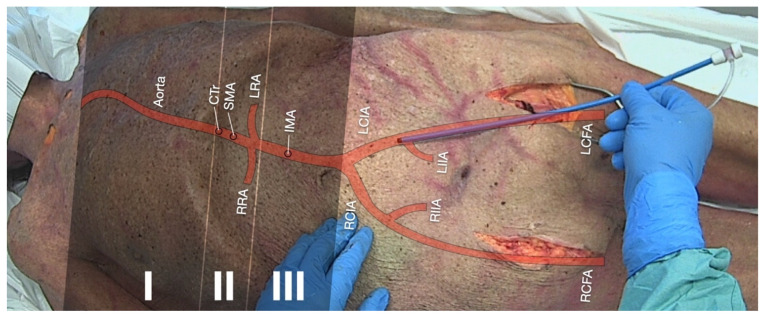
Schematic topographic projection of the aorta and its major branches on a cadaveric model: REBOA landing zones (**I**) (preferred in non-traumatic cardiac arrest), (**II**) (contraindicated), and (**III**) (preferred in pelvic trauma) are highlighted. Both femoral arteries have been prepared via a cutdown approach. Abbreviations: CTr = coeliac trunk, SMA = superior mesenteric artery, RRA = right renal artery, LRA = left renal artery, IMA = inferior mesenteric artery, RCIA = right common iliac artery, LCIA = left common iliac artery, RIIA = right internal iliac artery, LIIA = left internal iliac artery, RCFA = right common femoral artery, and LCFA = left common femoral artery.

In order to determinate the target area where the aortic occlusion balloon is supposed to be deployed, the REBOA concept defines specific “landing zones” (Figure 2) depending on the indication [94].

(1) The ascendant aorta with the aortic arch is occasionally referred to as “Zone 0”. Due to the branches of the carotid arteries supplying the brain with blood, balloon deployment is contraindicated in this zone.
(2) Zone I: The left subclavian artery to the celiac trunk. This position is preferred in non-traumatic cardiac arrest to enhance coronary and cerebral perfusion. Among other scopes of application, there may be aortic dissection or uncontrollable thoracic or visceral bleeding.
(3) Zone II: The coeliac trunk to the lowest renal artery supplying major intra- and retroperitoneal organs with blood. Balloon deployment is contraindicated in this zone.
(4) Zone III: The lowest renal artery to the aortic bifurcation. This position is preferred in uncontrollable sub-/pelvic or groin bleeding.

#### 3.5.1. Achieving Hemostasis in Severe Trauma and Traumatic Cardiac Arrest (TCA)

As previously stated, REBOA is used to control bleeding from non-compressible injuries in critically injured casualties with uncontrolled hemorrhagic shock unresponsive to volume therapy [93]. Depending on the underlying trauma, the balloon occlusion may be deployed either in landing zone I (thoracic and upper abdominal bleeding and aortic dissection) or III (sub-/pelvic or grain bleeding) (Figure 2) [94].

As this special issue’s focus is on sudden cardiac death rather than TCA, the application of REBOA in catastrophic bleeding is only briefly mentioned due to historic and didactic reasons. Pertinent literature might be of interest for further reading [93,94,95].

#### 3.5.2. Improving Coronary Perfusion during CPR

When applied in trauma patients, the aortic occlusion substantially increased blood pressure which was expected to result in increased cerebral and coronary perfusion. Consistently, it is alleged that the REBOA procedure might have an epinephrine-like effect on aortic and subsequently coronary pressures [10]. As previously stated, those are associated with improved rates of ROSC and survival [17]. In order to enhance cardiac and cerebral perfusion pressures during CPR, the REBOA catheter should be placed in landing zone I (Figure 2).

As a matter of fact, the first experimental studies confirmed enhanced coronary and cerebral perfusion after performing the REBOA procedure in a porcine cardiac arrest model [96]. On this occasion, the effects on both perfusion pressures and ROSC are comparable to those achieved from the application of epinephrine [96]. For a long time, merely case reports and observational studies were published reporting that REBOA is feasible and effective in non-trauma cardiac arrest [97,98,99,100,101,102,103,104]. The clinical studies predominantly lack control groups and are limited to surrogate outcomes, but support the physiological considerations on perfusion as previously described [102,103,104,105]. Hence, REBOA is increasingly seen as an adjunct to non-trauma advanced cardiac life support (Table 4). If performed rapidly by a highly skilled team from an experienced center, enabling a short collapse-to-balloon time, it might serve as a bridge to treatment until PCI or ECMO [106].

In a randomized controlled feasibility study, a team from Heidelberg University Hospital examines the value of a REBOA device in non-trauma cardiac arrest. In addition to its safety and performance, secondary outcome measures and surrogate parameters such as blood pressure, CPR time intervals, and ROSC are of the investigators’ particular interest [NCT05146661]. The currently ongoing multi-center randomized controlled REBOARREST trial is supposed to provide insight into survival, hemodynamics, organ function, and adverse events [107].

### 3.6. Arterial Blood Gas (ABG) Analysis

Point-of-care testing (POCT), similar to ABG analysis, is widely used in both intensive care medicine and emergency departments, as the knowledge of particular blood parameters may require further therapeutic procedures in the treatment of life-threatening conditions. Therefore, it is considered a common adjunct to the treatment of in-hospital cardiac arrest [5,54]. Beyond differentiating respiratory disorders, further, potentially reversible causes of cardiac arrest may be detected and treated by means of ABG analysis; for example, metabolic disorders, such as acidosis and alkalosis, as well as electrolyte abnormalities, such as hypo or hyperkalemia. The measurement results may trigger interventions such as the optimization of artificial respiration, the correction of acidosis (ventilatory or sodium bicarbonate), antidote, vasoactive, or fluid therapy, or may accelerate admission to the hospital for resuscitative hemodialysis [108,109].

Due to the size, energy demand, and characteristics of the analytical equipment, even if measured at the “point of care”, most POCT applications had been restricted to health care facilities. With the advent of portable devices, blood gas analyzers have become available in the field [110,111]. Meanwhile, technological advances have enabled a rapid and reliable application in the prehospital environment [108].

The parameters obtained may support the responsible team in diagnosis and treatment decision-making [108,109]. While survival data from RCTs are still missing, it seems obvious that patients might profit from the earliest possible recognition and treatment of reversible causes during OHCA [112].

### 3.7. Thoracic Decompression

In addition to chest trauma as a main cause, pulmonary diseases, such as acute exacerbation of chronic obstructive pulmonary disease with consecutive pneumothorax, may be accompanied by progressive ventilatory and/or cardiocirculatory distress [113]. There is a risk of developing a life-threatening tension pneumothorax, which can be fatal if left untreated. Needle decompression and thoracostomy with or without drainage insertion are, therefore, life-saving interventions. The relief of tension pneumothorax by means of pleural puncture or minithoracostomy is an established invasive procedure. It is one of the basic techniques in the care of critically ill patients and every doctor working in emergency medicine must be able to perform it [92,114].

**Figure 3:**
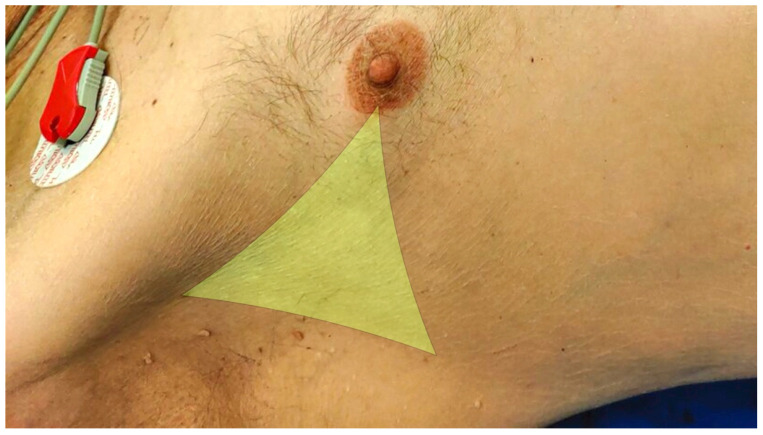
Projection of the Triangle of Safety formed by the posterior border of the pectoralis major muscle, the anterior border of the latissimus dorsi muscle, and the intermammillary line.

In highly dynamic situations with foudroyant shock, needle decompression is useful, as it is quick and easy to perform. Classically, it is performed in the Monaldi position in the second or third intercostal space of the midclavicular line. The indwelling venous cannulae often used for needle decompression are too short to reach the pleural space for a relevant proportion of patients [115,116]. For this reason, relevant course formats alternatively recommend needle decompression in the Bülau position in the fourth or fifth intercostal space between the anterior and midaxillary line (Figure 3) [117,118].

A tension pneumothorax can usually only be relieved for a short time with the puncture, so it seems sensible to always perform a minithoracostomy afterward [119]. The minithoracostomy is performed in the Bülau position. The life-saving intervention is the opening of the pleural space, not the insertion of a drain. Whether a chest drain should be inserted at the scene or in the hospital remains the subject of ongoing debate. Recent retrospective data suggest that there could be an increased risk of recurrent tension physiology if thoracostomy is not followed by the placement of a drain [120].

### 3.8. Pericardiocentesis

While chronic pericardial effusion, for example, due to infectious, inflammatory, or malignant disease, usually develops slowly, rapid accumulation in the event of a disease flare-up can lead to subacute cardiocirculatory decompensation. In the event of aortic dissection, after thoracic trauma or iatrogenic perforation (for example, PCI, aortic, or cardiac surgery), the development is dramatic. Even small amounts of blood can lead to a significant increase in intrapericardial pressure within a few minutes and clinically manifest pericardial tamponade [121].

**Figure 4:**
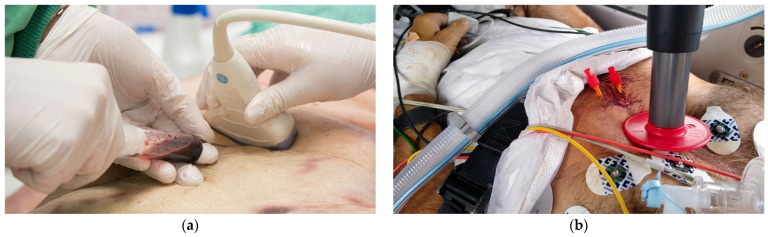
(**a**) Ultrasound-guided pericardiocentesis in a cadaveric model: The convex-array transducer is placed subxiphoidal to obtain a subcostal window 4-chamber view (S4CH) in order to guide the needle precisely under sight and thus achieve higher success rates; (**b**) Helicopter transport of a patient in sustained return of spontaneous circulation (ROSC) after ultrasound-guided relief of pericardial effusion in the field by the catheter-over-needle technique. Catheters have been left in place. An automated mechanical cardiopulmonary resuscitation (mCPR) device has been applied precautionarily.

Clinical diagnosis of pericardial tamponade (Beck’s Triad: hypotension with a narrowed pulse pressure, jugular venous distention, and muffled heart sounds) is difficult and unreliable, especially in a peri-arrest situation. As acute tamponade is not associated with a large effusion volume, a rather small effusion of 50 mL can cause cardiac tamponade in a hypovolemic state and lead to cardiac arrest. This reversible cause has to be treated immediately in order to restore perfusion. In this case, prehospital emergency ultrasonography is essential [121,122]. Furthermore, the integration of ultrasound reduces the time until relief of the pericardial tamponade (Figure 4a,b) [123].

In principle, two phenotypes of tamponade can be distinguished sonographically. On the one hand, a liquid tamponade is easily accessible for puncture; on the other hand, extensive clot formation can occur in the case of a hemopericardium. In the latter case, relief by pericardiocentesis is not promising and relief must be achieved by thoracotomy, as described below [54,119,121].

It must be mentioned restrictively that, to date, there is no systematic data available regarding the cause of cardiac tamponade in non-traumatic cardiac arrest. If the cardiac arrest was caused by aortic dissection or ventricular rupture, simply relieving tamponade could not solve the problem, as the underlying pathology cannot be treated with the options available in an out-of-hospital setting.

### 3.9. Resuscitative Thoracotomy

The resuscitative opening of the chest by means of a clamshell thoracotomy provides a quick overview and allows control of intrathoracic bleeding, proximal aortic compression, and access to the pericardium in cases of suspected or ultrasound-confirmed tamponade [92,124,125]. Therefore, it is indicated in traumatic cardiac arrest rather than sudden cardiac death. Nevertheless, this procedure is supposed to be discussed here as an advanced invasive resuscitation technique for the sake of completeness. As an invasive emergency technique, thoracotomy is firmly established in the current guidelines for cardiopulmonary resuscitation and polytrauma care, although the indication must be restrictive. According to the “4 E” rule, clamshell thoracotomy is only indicated if certain conditions concerning expertise, equipment, environment, and elapsed time are met [54].

**Figure 5:**
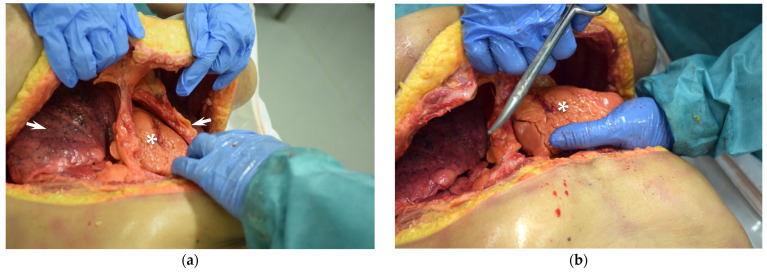
Clamshell thoracotomy in a cadaveric model: (**a**) Situs after thoracotomy, the chest is lifted by one rescuer to allow a good overview of the heart (asterisk) and lungs (arrowheads); (**b**) The second rescuer luxates the heart (asterisk) in order to be able to perform interventions.

Compared to left anterolateral thoracotomy, clamshell thoracotomy is more suitable for potentially reversible causes of trauma-induced cardiovascular arrest (Figure 5a,b). It allows an excellent overview of the intrathoracic organs and thus a wide range of interventions [124].

The pericardium is incised in a T-shape to relieve pericardial tamponade and to inspect the heart for treatable injuries. Pericardial tamponade can be cleared manually. Relevant injuries to the lung can be treated by clamping or hilum twists as a last resort. Subdiaphragmatic bleeding can be reduced by manual compression of the aorta against the spine [92]. If internal cardiac massage is required, the heart should be taken between both hands and compressed in a walking motion from the apex to the base about 80 times per minute. However, resuscitative thoracotomy does not make sense to perform a thoracotomy in order to be able to resuscitate an open heart [126]. Despite numerous impressive case reports from highly experienced centers, evidence for this highly invasive procedure from clinical trials under routine care conditions is lacking.

## 4. Discussion of Concepts and Strategies

**Figure 6:**
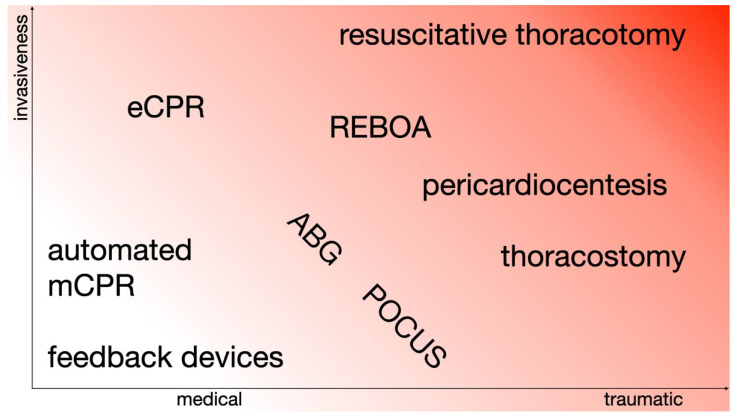
Advanced and invasive techniques may be considered in medical and traumatic cardiac arrest. Abbreviations: CPR = cardiopulmonary resuscitation, eCPR = extracorporeal CPR, mCPR = (automated) mechanical CPR, REBOA = resuscitative endovascular balloon occlusion of the aorta, ABG = arterial blood gas, and POCUS = point-of-care ultrasound.

With the increasing establishment of advanced and invasive techniques in ALS, procedures may now potentially be considered in medical circulatory arrest whose applications were previously limited to traumatic cardiac arrest or trauma life support. This could make the boundaries of indications appear increasingly blurred (Figure 6).

### 4.1. Decision-Making and Timing

Advanced and invasive techniques may have a positive impact on survival but can be fraught with technical, social, situational, and organizational hurdles. Therefore, it is important that the emergency crew knows the infrastructure, with its highly qualified and skilled teams, and can deploy resources that can respond to this time-critical emergency within minutes. Here, the geographical and health characteristics of the different urban and rural areas must also be considered [127].

Time plays a major role in the context of OHCA. Ideally, and in terms of survival with good neurological outcome, the low-flow time should not be greater than 60 min [128,129]. This presents a major challenge to emergency teams. A lot of information has to be filtered in a short time and a decision has to be made for or against a resource-intensive attempt to help. Even if the local conditions provide all technical and personnel resources, the process is significantly influenced by the decisions of the team on site. A predefined decision point or mental model can have a major impact on the stressful teamwork, the further course of resuscitation, and the survival of the patient.

For teamwork to succeed resiliently and function efficiently, mental models can be helpful. Emergency teams should use and implement mental models even before the emergency. This can establish a clear decision tree, streamline processes, and expedite decisions (for example, a mental model for refractory cardiopulmonary resuscitation in OHCA) [130].

The decision point is a formal team time-out to plan the next 60 min, maximize the use of team resources, and anticipate procedures. At this point, brief considerations should be verbalized in the scenario, decided interprofessionally, and initiated immediately.

Taking eCPR as an example, after the second rhythm analysis, or after intubation, the team decides loudly and unanimously that, based on the information available, eCPR is indicated and the cannulation team can be dispatched. As Tonna and colleagues conclude, due to limited data, much of the current practice is based on expert opinion and institutional knowledge, rather than scientific evidence [127]. Depending on the local strategy, whether the cannulation is in- or out-of-hospital, once reversible causes have been ruled out, preparation for cannulation or transport to the hospital follows. If the cannulation is attempted in-hospital, the decision to proceed with eCPR must be reconsidered by the receiving team [131]. As a result of rapid decision making, time-intensive steps can be taken early and conditions can be created for eCPR to begin in less than 60 min [132]. This example highlights the importance of timing in this process and how decision-making impacts patient survival.

Awareness of the right timing and early activation of advanced procedures are imperative to lead to the reduction in low-flow times and should be included in local standard operating procedures (SOPs). Mental models for rare events and a decision point as a trigger in the resuscitation algorithm could support the processes.

Before highly invasive techniques are carried out, a short team briefing should take place in order to give all those involved in the operation the opportunity to “take themselves out of the situation” if they fear that they will be too emotionally burdened by the procedures. After the response, a structured team debriefing is a valuable educational strategy to improve team performance [133]. While the effect of debriefing on long-term patient outcomes is uncertain, there is evidence that structured debriefing improves clinical education, the efficiency of work, team climate, and patient safety [134,135,136]. Teams with alternating compositions, which is typical for emergency medicine, benefit most from structured debriefing [136]. Especially after stressful emergency responses, a structured and—if necessary, moderated—reappraisal and debriefing should also be offered for all personnel involved in a protected setting [137]. Early signs and support for stress reactions should also be made a subject of discussion.

### 4.2. Emergency Response and Continuing Care Structures

A promising approach to bring the therapeutic options previously described to the roadside is rapid response vehicles staffed with an experienced emergency physician. There are some proven concepts, such as London’s air ambulance, which operates an advanced trauma team helicopter and a rapid response car during night time or in adverse weather or rather flight conditions [18]. Heidelberg University Hospital also operates a rapid response vehicle providing devices and experienced staff for invasive interventions, the “medical intervention car (MIC)” [19]. Utilizing both modes, rapid response cars and rescue helicopters, appears reasonable, as this concept will combine the advantages of a highly flexible system within the narrow spaces of a city with a system that allows for the deploying of the techniques quickly over long distances to rural areas.

Invasive procedures may be associated with relevant blood loss due to the intervention itself (e.g., ECMO and thoracotomy) or the underlying mechanism (e.g., trauma or aortic dissection) leading to cardiac arrest. In addition to bleeding control, volume replacement plays a central role. It is questionable whether a patient can be stabilized with crystalloid or colloid infusion solutions alone in the case of massive blood loss. While blood transfusion in an out-of-hospital setting was unimaginable in large parts of the world until recently, blood products are now increasingly available on the scene [19,54,138,139,140,141].

Embedding the algorithm on advanced and invasive procedures in local care structures is essential to ensure adequate continuing care for critically ill patients. This begins with raising public awareness programs for lay resuscitation. Receiving hospitals must have the necessary human, medical, and technical resources to ensure a seamless continuation of treatment and manage any complications that may arise, which is the case with cardiac arrest centers (CAC).

## 5. Conclusions

Despite all innovations, situation awareness, continuous training of technical and non-technical skills, and proper (effective and continuous) chest compressions, the earliest possible defibrillation and artificial respiration remain the basic framework of CPR.

Ultrasound under CPR is increasingly becoming a key skill for the evaluation of reversible causes of cardiac arrest, optimizing chest compressions and evaluating the hemodynamic situation after obtaining sustained ROSC.

For selected patient groups, under certain circumstances and, in particular, fields of application, advanced and invasive techniques may be an option to ensure sufficient organ (in particular, coronary and cerebral) perfusion. There is a lack of clear evidence regarding solid endpoints, such as survival. Hence, these techniques should only be conducted with clear therapeutic conceptions as a bridge to treatment by a specialist team.

This should be part of a larger treatment concept that requires appropriate emergency response systems, as well as definitive treatment in centers with established continuing care structures.

The decisions and timing made by the emergency team have a major impact on the time-critical scenario. Therefore, it is essential that emergency teams know and use local resources, establish mental models for rare emergencies, and set decision points as triggers that initiate processes early.

## Figures and Tables

**Table 1:** Overview of RCTs on real-time audio or audiovisual CPR feedback systems vs. conventional manual chest compression.

Trial Abbreviation and Quotation	Design and Inclusion Criteria	Main Findings and Limitations
Compression Feedback for Patients with In-hospital Cardiac Arrest [29]	Cardio First Angel^®^ vs. conventional manual CPR; prospective individualized RCT, multi-center (Iran); in-hospital; 2015; 450 vs. 450 cases out of 1454 Inclusion criteria: age ≥ 18 years, admitted to ICU, resuscitation status (full code), and informed consent	↑ ROSC, ↑ ICU discharge, ↑ hospital discharge Limitations: selection bias (no primary cardiac entity), performance bias (quality monitoring), and training effect
Automated Real-time Feedback on CPR Study [31]	HeartStart-MRx^®^ vs. conventional manual CPR; prospective cluster RCT, multi-center (USA, Canada); out-of-hospital; 2007–2009; 815 vs. 771 cases out of 1819 Inclusion criteria: age ≥ 20, defibrillation or chest compressions by study vehicle team and non-TCA	↑ hands-on time, ↑ compression depth, ↑ complete release, ± ROSC, ± admission, ± discharge, ± CPC Limitations: design (allocation concealment and no regression analysis), selection bias (trial vehicle), and training effect

Legend: ± = equal, comparable, and no statistically significant difference; ↑ = more, higher, better, and superior; ↓ = less, lower, worse, and inferior. Abbreviations: RCT = randomized controlled trial, CPR = cardiopulmonary resuscitation, ICU = intensive care unit, ROSC = return of spontaneous circulation, CPC = Glasgow–Pittsburgh cerebral performance category, and TCA = traumatic cardiac arrest.

**Table 4:** Overview of selected studies on REBOA in non-TCA.

Trial Abbreviation and Quotation	Design and Inclusion Criteria	Main Findings and Limitations
(a) RCTs on REBOA in non-traumatic cardiac arrest		
REBOARREST [107]	ALS an REBOA vs. standard ALS; RCT, multi-center (Norway); out-of-hospital; 2022-ongoing; calculated enrollment 200 patientsInclusion criteria: age 18–80, OHCA, non-TCA, witnessed or <10 min from the debut of arrest, and commenced ALS established and can be continued	Study currently recruiting
(b) observational trials on REBOA in non-traumatic cardiac arrest		
Feasibility of Pre-Hospital REBOA [98]	ALS an REBOA vs. standard ALS; RCT, single-center (Norway); out-of-hospital, helicopter; 2018–2019; 10 patientsInclusion criteria: age 18–75, OHCA, non-TCA, and witnessed or <10 min from the debut of arrest	The attempt was 100% successful (80% first attempt), 60% ROSC, 30% admission to hospital, 10% survival (30 days), procedural time 11.7 ± 3.2 min, and etCO_2_ + 1.75 kPa after 1 minLimitations: feasibility study, a decision by study group, single-center, a small number of researchers and patients, and no autopsies (adverse effects)
NEURESCUE^®^ Device as an Adjunct to Cardiac Arrest [NCT05146661]	REBOA device; interventional open-label single group study, single-center (Germany); in hospital emergency department; 2022-ongoing; calculated enrollment 10 patientsInclusion criteria: age 18–75, witnessed, CPR initiated ≤ 7 min of presumed arrest, not responding to standard ALS, and total CPR time ≤ 40 min at enrollment	Study currently recruiting

Legend: ± = equal, comparable and no statistically significant difference; ↑ = more, higher, better, and superior; ↓ = less, lower, worse, and inferior. Abbreviations: REBOA = resuscitative endovascular balloon occlusion of the aorta, TCA = traumatic cardiac arrest, OHCA = out-of-hospital cardiac arrest, RCT = randomized controlled trial, CPR = cardiopulmonary resuscitation, ALS = advanced life support, and etCO_2_ = end-tidal carbon dioxide partial pressure.

## Data Availability

Not applicable.

## Supplementary Materials

The following supporting information can be downloaded at https://www.mdpi.com/article/10.3390/jcm11247315/s1, Video S1: Identification of the area of maximal compression (AMC) during ongoing manual chest compressions. Table S1: Searched literature sources; Table S2: Search terms.

## Abbreviations

ABG	arterial blood gas
ALS	advanced life support
AMC	area of maximal compression
CAC	cardiac arrest center
CI	confidence interval
COI	conflict of interest
CPC	Glasgow–Pittsburgh cerebral performance category
CPR	cardiopulmonary resuscitation
CT	computed tomography
CTr	coeliac trunk
DSMB	data and safety monitoring board
DVT	deep vein thrombosis
ECLS	extracorporeal life support
ECMO	extracorporeal membrane oxygenation
eCPR	extracorporeal cardiopulmonary resuscitation (CPR)
eFAST	extended focused assessment with sonography for trauma
EMS	emergency medical service
etCO_2_	end-tidal carbon dioxide partial pressure
FDA	US Food and Drug Administration
FEEL	focused echocardiographic evaluation in life support
ICU	intensive care unit
IHCA	in-hospital cardiac arrest
IMA	inferior mesenteric artery
IVC	inferior vena cava
LCFA	left common femoral artery
LCIA	left common iliac artery
LIIA	left internal iliac artery
LRA	left renal artery
LV	left ventricle
LVOT	left ventricular outflow tract
mCPR	(automated) mechanical cardiopulmonary resuscitation (CPR)
ME Bicaval	midoesophageal bicaval view
ME4CH	midoesophageal 4-chamber view
MELAX	midoesophageal long axis view
MIC	medical intervention car, a physician-staffed rapid response vehicle
mRS	modified Rankin Scale
OHCA	out-of-hospital cardiac arrest
OR	odds ratio
PCI	percutaneous coronary intervention
PE	pulmonary embolism
PEA	pulseless electrical activity
PLAX	parasternal long-axis view
POCT	point-of-care testing
POCUS	point-of-care ultrasound
RACA	return of spontaneous circulation (ROSC) after cardiac arrest
RCFA	right common femoral artery
RCIA	right common iliac artery
RCT	randomized controlled trial
REBOA	resuscitative endovascular balloon occlusion of the aorta
RIIA	right internal iliac artery
ROSC	return of spontaneous circulation
RRA	right renal artery
RV	right ventricle
S4CH	subcostal window 4-chamber view
SMA	superior mesenteric artery
SOP	standard operating procedure
TAVI	transcatheter aortic valve implantation
TCA	traumatic cardiac arrest
TGSAX	transgastric short axis view
TOE	trans-oesophageal echocardiography
TTE	trans-thoracic echocardiography
VF	ventricular fibrillation
