# Supplementary material for: Advanced and Invasive Cardiopulmonary Resuscitation (CPR) Techniques as an Adjunct to Advanced Cardiac Life Support
Source: J Clin Med. 2022 Dec 9;11(24):7315. doi: 10.3390/jcm11247315 (PMC9781548; doi:10.3390/jcm11247315)
Supplement: Supplementary file 1 [file jcm-11-07315-s001.zip › supplementary file 2022-11-25.pdf]

Journal of
Clinical Medicine

MDPI

Supplementary material

Advanced and invasive Cardiopulmonary Resuscitation (CPR) Techniques as an Adjunct to Advanced Cardiac Life Support

Manuel Obermaier *, Stephan Katzenschlager, Othmar Kofler, Frank Weilbacher and Erik Popp

Search strategy

Table S1. Searched literature sources.

Source	Search engine or database
**Scientific databases**	PubMed® MEDLINE<br>Cochrane library<br>Clarivate Web of Science™<br>Elsevier Embase®<br>Elsevier Scopus®
**Preprint servers**	PrePubMed<br>(containing: arXiv q-bio, PeerJ Preprints, bioRxiv, F1000Research, preprints.org, The Winnower, Nature Precedings, Wellcome Open)<br>medRxiv<br>ResearchGate
**Clinical trials registries**	International Clinical Trials Registry Platform (ICTRP)<br>(containing: Australian New Zealand Clinical Trials Registry, Chinese Clinical Trial Registry, ClinicalTrials.gov, EU Clinical Trials Register (EU-CTR), ISRCTN, The Netherlands National Trial Register, Brazilian Clinical Trials Registry (ReBec), Clinical Trials Registry - India, Clinical Research Information Service - Republic of Korea, Cuban Public Registry of Clinical Trials, German Clinical Trials Register, Iranian Registry of Clinical Trials, Japan Registry of Clinical Trials (jRCT), Pan African Clinical Trial Registry, Sri Lanka Clinical Trials Registry, Thai Clinical Trials Registry (TCTR), Peruvian Clinical Trials Registry (REPEC), Lebanese Clinical Trials Registry (LBCTR))
**Guideline sources**	European Resuscitation Council (ERC)<br>International Liaison Committee on Resuscitation (ILCOR)<br>Arbeitsgemeinschaft der Wissenschaftlichen Medizinischen Fachgesellschaften (AWMF)
**Internet search engines**	Google

Table S2. Search terms.

General	Advanced techniques	Invasive procedures
cardiac arrest	techniques	procedures
sudden heart death	real-time feedback	extracorporeal CPR
out-of-hospital cardiac arrest	chest compression devices	ECLS
heart massage	mechanical CPR	va-ECMO
circulation	automated CPR	REBOA
	ultrasound	pericardial puncture
	sonography	pericardiocentesis
	POCUS	thoracostomy
	arterial blood gas	clamshell
	device	resuscitative thoracotomy
